# Effect of *Pediococcus pentosaceus* DB12 Cell-Free Supernatant on Controlling *Aspergillus flavus* Contamination in Postharvest Fresh Peanuts

**DOI:** 10.3390/foods15213939

**Published:** 2026-11-03

**Authors:** Jiake Guan, Haoxin Lv, Liang Chen, Xiangzhen Yin, Honglei Zhu, Beibei Xie, Can Cui, Yan Zhao, Yanfei Li

**Affiliations:** 1 School of Food and Strategic Reserves, Henan University of Technology, Zhengzhou 450001, China; 18237275759@163.com (J.G.); chenliang@haut.edu.cn (L.C.); 19950308400@163.com (H.Z.); 18637167182@163.com (B.X.); cc13526618922@163.com (C.C.); zhaoyanss10@126.com (Y.Z.); liyanfei@haut.edu.cn (Y.L.); 2 Peanut Quality Breeding Laboratory, Shandong Peanut Research Institute, Qingdao 266100, China; yinxiangzhen1985@163.com

**Keywords:** fresh peanut, *Aspergillus flavus*, contamination, *Pediococcus pentosaceus* DB12 cell-free supernatant, biocontrol agent

## Abstract

Fresh peanuts (*Arachis hypogaea* L.) are highly susceptible to *Aspergillus flavus* contamination, leading to rapid quality deterioration. Lactic acid bacteria (LAB) are well established as natural agents for preserving agricultural products and inhibiting pathogenic microorganisms. However, their efficacy against *A. flavus* in fresh peanuts remains underexplored. This study evaluated the efficacy of *Pediococcus pentosaceus* DB12 cell-free supernatant (*P. pentosaceus* DB12 CFS) in mitigating *A. flavus* contamination in fresh peanuts, integrating assessments of quality parameters, microbial indices and untargeted metabolomics. Results demonstrated that *P. pentosaceus* DB12 CFS effectively mitigated *A. flavus*-induced deterioration in fresh peanuts, as evidenced by improved retention of color, textural hardness, and weight during storage. *P. pentosaceus* DB12 CFS also significantly reduced total number of mold colonies and mildew rate. Furthermore, it suppressed electrolyte leakage and malondialdehyde accumulation, inhibited peroxide and acid value elevation, and minimized the degradation of soluble sugars and sucrose. Metabolomic analysis further revealed the activation of phenylalanine metabolism, stilbenoid, diarylheptanoid and gingerol biosynthesis, and glutathione metabolism, thereby enhancing disease resistance in fresh peanuts. This study not only establishes the efficacy of *P. pentosaceus* DB12 CFS in mitigating postharvest *A. flavus* contamination in fresh peanuts but also provides a foundation for developing safe and efficient biocontrol technologies.

## 1. Introduction

As an annual legume crop with its origin in South America, the peanut (*Arachis hypogaea* L.) holds significant status as a primary oilseed crop. Its cultivation extends to approximately 120 countries globally [1]. Global peanut production, measured on a kernel basis, exceeded 54.23 million metric tons in 2023, with continued growth projected for the industry [2]. Currently, most of the harvested peanuts are utilized for oil extraction or dried snack production [3]. However, with substantial improvements in living standards, consumers increasingly seek higher nutritional quality and superior sensory attributes [4]. Fresh peanuts, which are consumed raw or boiled without undergoing desiccation, optimally preserve their sensory characteristics, nutritional value, and health-promoting properties, thereby overcoming the nutritional degradation commonly associated with thermally processed alternatives such as roasted or fried peanuts [5].

Peanuts are highly prone to *Aspergillus* spp. infection during postharvest handling, storage, and distribution as a consequence of their high moisture content and nutrient-rich profile [6,7]. During colonization by *Aspergillus flavus*, severe sensory deterioration occurs, characterized by dull discoloration, the appearance of yellow-green colonies on the surface, and the development of strong musty odors. Concurrently, the proliferation of fungal hyphae compromises tissue integrity, causing texture softening and substantial nutrient depletion [8,9]. As *A. flavus* continues to proliferate, the aflatoxins it produces pose a serious threat to human health [10,11]. These visible quality impairments often result in the rejection or disposal of fresh peanuts at multiple points within the logistical sequence, thereby causing considerable economic losses [12,13,14]. Therefore, it is imperative to implement effective strategies to control postharvest *A. flavus* contamination in fresh peanuts to maintain product quality throughout storage, transportation, and marketing phases.

Current research has investigated various approaches to mitigate *A. flavus* contamination in peanuts, encompassing traditional methods such as desiccation and the application of chemical fungicides, as well as emerging innovations in packaging materials [15,16,17]. Pulsed light technology demonstrates effective inactivation of *A. flavus* spores in peanuts, thereby reducing fungal infection and suppressing aflatoxin B_1_ synthesis [18]. 2-ethylhexanol vapor disrupts the cell envelope of *A. flavus* to inhibit spore germination [19]. The application of thymol essential oil to stored peanuts effectively suppresses *A. flavus* colonization and limits aflatoxin accumulation [20]. Furthermore, the chitosan/cinnamaldehyde edible composite coating inhibits *A. flavus* growth by forming a semi-permeable barrier and releasing antifungal active ingredients. Besides suppressing fungal development, this coating can also alleviate insect harm while conserving seed germination capability and seedling vigour [21]. However, existing strategies are associated with limitations such as residual chemical contamination, high operational costs, and insufficient efficacy [22]. Therefore, the development of environmentally friendly, safe, and efficient bio-antifungal agents is of critical importance for the effective control of postharvest *A. flavus* contamination in fresh peanuts.

Biocontrol agents offer advantages in terms of cost-effectiveness, safety, and high efficacy, making them promising alternatives to conventional preservation methods [23]. As bioprotective agents, lactic acid bacteria (LAB) could be utilized in the food manufacturing sector owing to their capacity to generate antimicrobial substances [24]. The cell-free supernatant (CFS) of LAB contains a mixture of these metabolites, showing potential inhibitory activity against common food spoilage microorganisms [25,26]. Recent studies demonstrate that *Weissella cibaria* DA2 CFS effectively delays quality deterioration in sweet corn kernels during postharvest storage [27], while *Lactiplantibacillus pentosus* CW5 CFS effectively maintains postharvest quality in kiwifruit by delaying softening and suppressing microbial decay [28]. In addition, *Pediococcus pentosaceus* (*P. pentosaceus*) strains show the ability to inhibit *A. flavus* contamination in food materials [29], which makes it possible to apply LAB CFS to suppress *A. flavus* contamination in fresh peanuts after harvest.

This study assessed the efficacy of *P. pentosaceus* DB12 CFS in mitigating postharvest *A. flavus* contamination in fresh peanuts through a multidimensional quality evaluation. The protective effects were quantified using storage stability indices, membrane integrity degradation, oxidative rancidity of peanut oil, and reductions in starch, soluble sugar and sucrose content. Complementary untargeted metabolomics analysis provided insights into the mechanisms underlying the biocontrol activity mediated by *P. pentosaceus* DB12 CFS. This study aims to establish a mechanistic foundation for the application of LAB CFS in preventing *A. flavus*-induced postharvest quality deterioration in fresh peanuts.

## 2. Materials and Methods

### 2.1. Materials

“Dabaisha 171” peanuts were harvested in 2024 from Xinzheng City (34°32′16″ N, 113°49′38″ E), Henan Province, China. *P. pentosaceus* DB12 (GenBank accession number of *16S rRNA* gene: OR084832) was isolated by our research team and preserved at −80 °C. Toxigenic *A. flavus* 3.395 was provided by the China General Microbiological Culture Collection Center.

### 2.2. Fresh Peanut Processing

#### 2.2.1. *P. pentosaceus* DB12 CFS Preparation

Based on the method of Wang et al. [27], *P. pentosaceus* DB12 CFS was prepared. *P. pentosaceus* DB12 strain was grown under anaerobic conditions at 37 °C for 48 h, utilizing Man–Rogosa–Sharpe (MRS) agar as the growth medium. Single colonies were subsequently inoculated into MRS broth using an inoculation loop and incubated at 37 °C under shaking conditions (200 rpm) for 48 h. Following two consecutive subcultures, bacterial suspensions (OD_600_ = 1.37) were centrifuged at 4 °C and 8000× *g* for 10 min. The resulting supernatant was collected and filtered through a 0.22 μm sterile membrane. The sterility of filtrate was verified by spreading the supernatant on MRS agar plates; no bacterial colonies were observed after incubation, confirming the absence of viable bacterial cells. The prepared CFS was stored at 4 °C for later use.

#### 2.2.2. *A. flavus* Spore Suspension Preparation

*A. flavus* 3.395 strain was incubated on potato dextrose agar (PDA) at 28 °C for three days to produce spores. Subsequently, the spores were harvested from the PDA surface by washing with an appropriate volume of sterile distilled water. The obtained spore suspension was vortexed sufficiently and then adjusted to attain a final cell density of 5 × 10^5^ CFU/mL [30].

#### 2.2.3. Peanut Sample Processing

Fresh peanut samples were processed according to the methodology outlined by Lv et al. [31] with small alterations. Peanuts with full and uniform size (moisture content: 40.3% ± 1%, *w*/*w*) were selected and surface-sterilized using a 0.3% (*w*/*w*) sodium hypochlorite solution, rinsed with distilled water, and ventilated at 25 ± 1 °C. Following ventilation and drying, an aflatoxin-producing spore solution was evenly sprayed onto the peanut surfaces at a rate of 5 mL/kg and allowed to air-dry for 15 min. Subsequently, 38 mL/kg of *P. pentosaceus* DB12 CFS was evenly applied to the fresh edible peanut pods, and the treated samples were transferred to an incubation environment maintained at 25 ± 1 °C for further ventilation and drying over a 30-min period. Fresh peanuts with pod shells treated with *P. pentosaceus* DB12 CFS were designated as the treatment group (CFS group); meanwhile, samples receiving an equivalent volume of sterile distilled water were designated as the blank control group (CK group). All samples were stored under controlled temperature conditions (4 ± 0.3 °C), and sampling was conducted at 6-day intervals throughout the experimental period.

### 2.3. Assessment of Color, Hardness and Weight Loss Ratio in Fresh Peanuts

The color measurement method of fresh peanuts was based on Peng et al. [32] with small alterations. A Konica Minolta CR-410 colorimeter (Konica Minolta Co., Ltd., Tokyo, Japan) was applied to acquire L*, a* and b* readings from fresh peanut shells and kernels. Five parallel samples were set up for each set of experiments.

The hardness measurement method for fresh peanuts followed Wu et al. [5], with minor adjustments. Fresh peanuts with shells were randomly sampled from all groups, and particles were placed in a texture analyzer (TA-XT Plus, Stable Micro System Inc., London, UK). Five parallel samples were set up for each set of experiments, and all results were expressed as N.

The measurement method for the weight loss ratio of fresh peanut kernels was adapted from Qian et al. [33] with slight modifications. Ten peanuts were randomly picked up from each group and weighed, and all results were expressed as %. Following formula was used to calculate the weight loss ratio:

*Weight loss ratio* (%) = (*w*_0_ − *w*_1_)/*w*_0_ × 100% (1)

where w_0_ denotes the initial weight of the peanut kernels at the beginning of the experiment, and w_1_ denotes the weight measured on the current observation date.

### 2.4. Determination of the Total Number of Mold Colonies and Mildew Rate of Fresh Peanuts

The total number of mold colonies was counted based on the method of Zhang et al. [34]. Briefly, the peanut kernels were homogenized with sterile water and shaken vigorously to prepare a suspension with a dilution factor of 10^−1^. Subsequently, suspensions with a dilution factor of 10^−2^ and 10^−3^ were prepared in sequence. Then, 100 μL of each suspension at different dilution levels was transferred onto potato dextrose agar (PDA) medium, spread evenly, and incubated in a constant-temperature incubator at 28 °C for 3 to 5 days. Finally, plates containing 10 to 150 CFU were selected, and the colonies were enumerated to determine the total mold colony count. Three biological replicates were prepared for each dilution gradient, and the results were expressed as the logarithm of total colony counts (lg CFU/g).

The mildew rate was calculated following the method of Liu et al. [35]. Fifty peanut pods were randomly selected for mildew rate assessment. Samples exhibiting visible mold colonies were classified as mold-infected, and all results were expressed as %. The mildew rate was calculated based on the following formula:

*Mildew rate* (%) = *w*_1_/*w*_0_ × 100% (2)

where w_0_ denotes the total number of peanut pods, and w_1_ denotes the number of mold-infected peanut pods.

### 2.5. Determination of Conductivity and Malondialdehyde (MDA) Content in Fresh Peanuts

The conductivity was detected with the method described by Zhao et al. [36]. Ten undamaged peanut kernels, each weighing 11.3 ± 0.5 g, were selected and subjected to triple rinsing with deionized water, followed by blotting dry with filter paper. Subsequently, the samples were placed into a conical flask with 50 mL distilled water. After sealing with plastic wrap, they were maintained at 25 ± 0.3 °C for 24 h. A conical flask with 50 mL distilled water served as the control. All results were expressed as μS/cm/g. The conductivity was calculated based on the following formula:

*Conductivity* (μS/cm/g) = (*C*_1_ − *C*_0_)/*M* (3)

where C_1_ denotes the conductivity of peanut kernel extract solution, C_0_ denotes the conductivity of water, and M denotes the weight of peanut kernel sample.

According to Wu et al. [5], the MDA content was assessed. Around 1 g frozen peanut kernel powder was homogenized in 5.0 mL 10% (*w*/*v*) TCA solution. The mixture was centrifuged at 4 °C for 10 min with 8000× *g*. Following this, 2.0 mL supernatant was mixed with equal volume of 0.6% (*w*/*v*) TBA. The resulting mixture was incubated in a water bath at 100 °C for 15 min. After that, they were promptly cooled and then centrifuged at 10,000× *g* for 10 min at 4 °C. Absorbance at 450, 532, and 600 nm was measured using the collected supernatant. All results were expressed as μmol/kg.

### 2.6. Determination of Peroxide and Acid Values in Fresh Peanuts

Crude peanut oil was extracted via cold pressing with petroleum ether, following a modified method of Chen et al. [37]. Peroxide value and acid value were determined according to the procedure described by Dai et al. [38]. For peroxide value measurement, 3 g of oil was dissolved in a chloroform–acetic acid mixture, treated with saturated KI solution, and titrated with Na_2_S_2_O_3_ after adding starch indicator. For acid value, 20 g of oil was extracted with a diethyl ether–isopropanol mixture and titrated with standardized NaOH solution using phenolphthalein as the indicator. The peroxide value was expressed as mmol/kg, whereas the acid value was expressed as mg/g.

### 2.7. Determination of Starch, Soluble Sugar and Sucrose Content in Fresh Peanuts

The starch content, soluble sugar content and sucrose content were measured using the following three assay kits from Beijing Solarbio Science & Technology Co., Ltd. (Beijing, China): the Plant Starch Content Assay Kit (BC6500), Soluble Sugar Content Assay Kit (BC0035), and Sucrose Content Assay Kit (BC6930), respectively. Experiments were conducted following the protocol of Ren et al. [39] with slight adjustments. All results were expressed as mg/g.

### 2.8. Metabolomic Analysis

Metabolites were extracted from fresh peanuts following the protocol established by Qu et al. [40] and analyzed using liquid chromatography–mass spectrometry (LC-MS/MS). Samples (50 mg each) from 0 d, CK-24 d, and CFS-24 d were homogenized with 1000 μL extraction solvent (methanol: acetonitrile: water = 2:2:1, *v*/*v*), supplemented with an internal standard (20 mg/L). Following the addition of steel beads, the samples were vortexed, extracted, centrifuged, and transferred to autosampler vials for instrumental analysis. Each group included six biological replicates.

The LC-MS/MS metabolomic analysis was performed on a Waters Acquity I-Class PLUS (Waters Corporation, Milford, MA, USA) ultra-high performance liquid chromatograph coupled to a Waters Xevo G2-XS QTof high-resolution mass spectrometer (Waters Corporation, Milford, MA, USA). Chromatographic separation was carried out using a Waters Acquity UPLC HSS T3 column (1.8 μm, 2.1 × 100 mm). For both positive and negative ion modes, mobile phase A was 0.1% formic acid aqueous solution, and mobile phase B was 0.1% formic acid acetonitrile. The injection volume was 2 μL. Mass spectrum acquisition was operated in MSE mode controlled by MassLynx V4.2 software (Waters). Dual-channel data acquisition was performed at low and high collision energy simultaneously. The low collision energy was off, and the high collision energy ranged from 10–40 V. The scanning frequency was 0.2 s per mass spectrum. ESI source parameters were set as follows: capillary voltage of 2500 V (positive ion mode) or −2000 V (negative ion mode); cone voltage of 30 V; ion source temperature of 100 °C; desolvent gas temperature of 500 °C; backflush gas flow rate of 50 L/h; and desolventizing gas flow rate of 800 L/h. In this MSE acquisition mode, precursor ions and their corresponding product fragment ions were simultaneously acquired for all detected metabolites to support compound identification.

Principal Component Analysis (PCA) and Orthogonal Partial Least Squares Discriminant Analysis (OPLS-DA) were conducted on the acquired metabolomic profiles. Differentially accumulated metabolites (DAMs) were then identified, hierarchically clustered, and functionally annotated through metabolic pathway analysis.

### 2.9. Statistical Analysis

All experimental measurements were performed with three biological replicates unless otherwise stated. The data are presented as the average values ± standard deviation. Significant differences between CK and CFS groups at each time point were assessed by one-way ANOVA with Duncan’s post hoc test (*p* < 0.05). Statistical analyses were performed in Microsoft Excel 2019; all figures displaying physiological and microbiological findings were generated in Origin 2026.

The peanut metabolomics experiments using LC-MS/MS were performed six times in parallel. Raw data were acquired using MassLynx V4.2 software. Principal component analysis (PCA) and orthogonal partial least squares-discriminant analysis (OPLS-DA) were performed with the software package of Biomarker Technologies. In addition, metabolomics graphs were created using the online bioinformatics platform Biocloud (https://www.biomarker.com.cn/).

## 3. Results

### 3.1. Alterations in Color, Hardness and Weight Loss Rate

**Figure 1:**
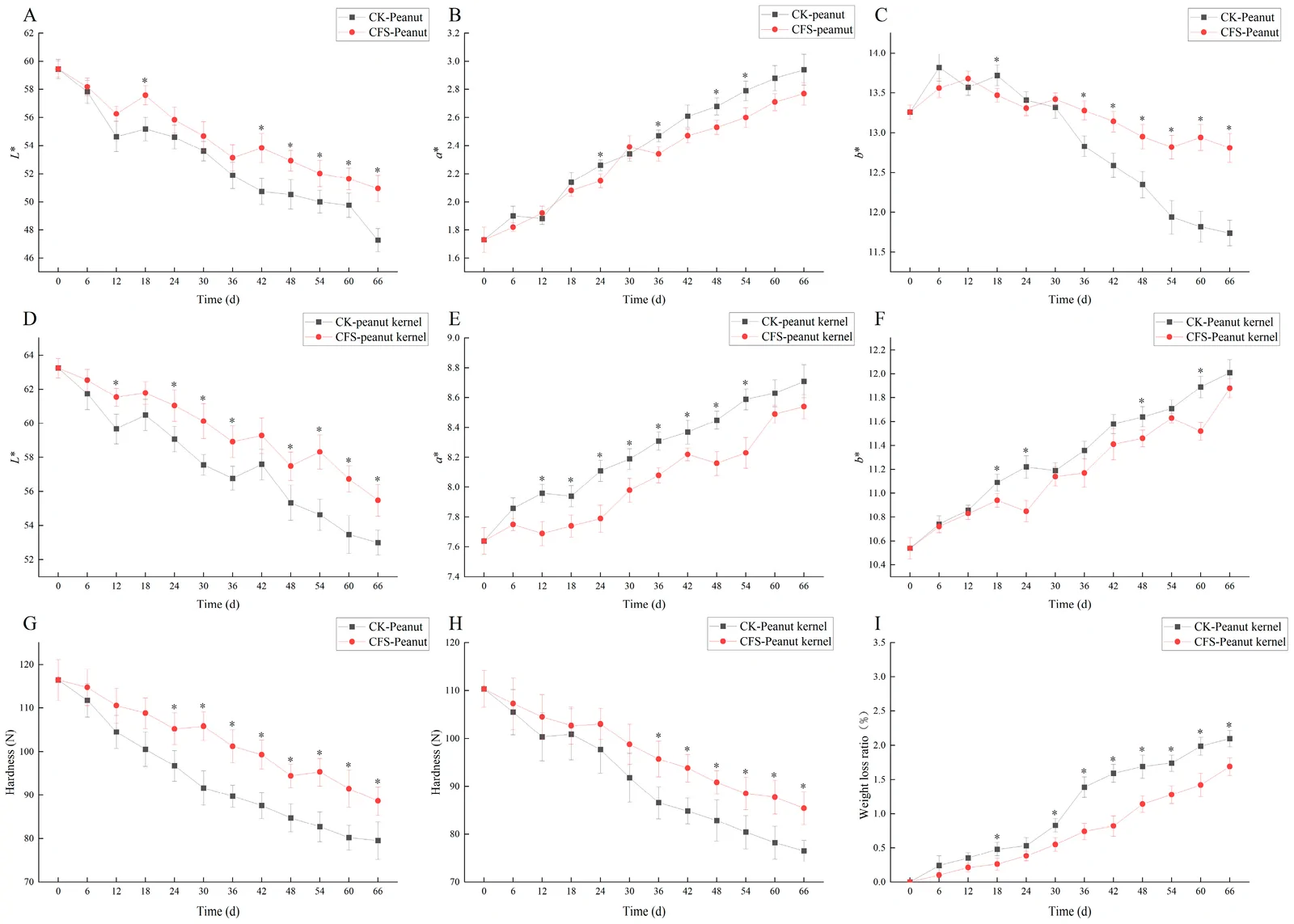
Effects of *Pediococcus pentosaceus* DB12 on color parameters, hardness, and weight loss ratio of fresh peanuts during storage. (**A**–**C**) L*, a* and b* values of fresh peanuts, respectively. (**D**–**F**) L*, a* and b* values of peanuts, respectively. (**G**) Hardness of fresh peanuts. (**H**) Hardness of peanut kernels. (**I**) Weight loss ratio of peanut kernels. CFS: fresh peanuts treated with *P. pentosaceus* DB12 CFS; CK: fresh peanuts in the control group. Note: asterisks (*) indicate statistically significant differences (*p* < 0.05) between the CK and CFS groups.

Color changes of fresh peanuts are shown in Figure 1A–C. The L* and b* values of peanut pods exhibited a declining trend, with significantly higher values observed in the CFS group. In contrast, peanut pods exhibited an upward trend in a* value, with no significant difference between the CFS and CK groups. Regarding peanut kernels, their color changes are illustrated in Figure 1D–F. The L* value of the kernels decreased over time, with the CFS group showing significantly higher L* values than the CK group. Meanwhile, both the a* and b* values of peanut kernels demonstrated an upward trend, with significantly lower values recorded in the CFS group.

Hardness changes of fresh peanuts and kernels are shown in Figure 1G,H. During storage, both peanut pods and kernels demonstrated a declining trend in hardness, with the CFS group showing significantly higher hardness values relative to the CK group.

As shown in Figure 1I, both peanut groups showed a progressive increase in weight loss ratio during the entire storage period. Relative to the CK group, the CFS group demonstrated a statistically significant difference in weight loss ratio starting from day 30.

### 3.2. Changes in the Total Number of Mold Colonies and Mildew Rate

**Figure 2:**
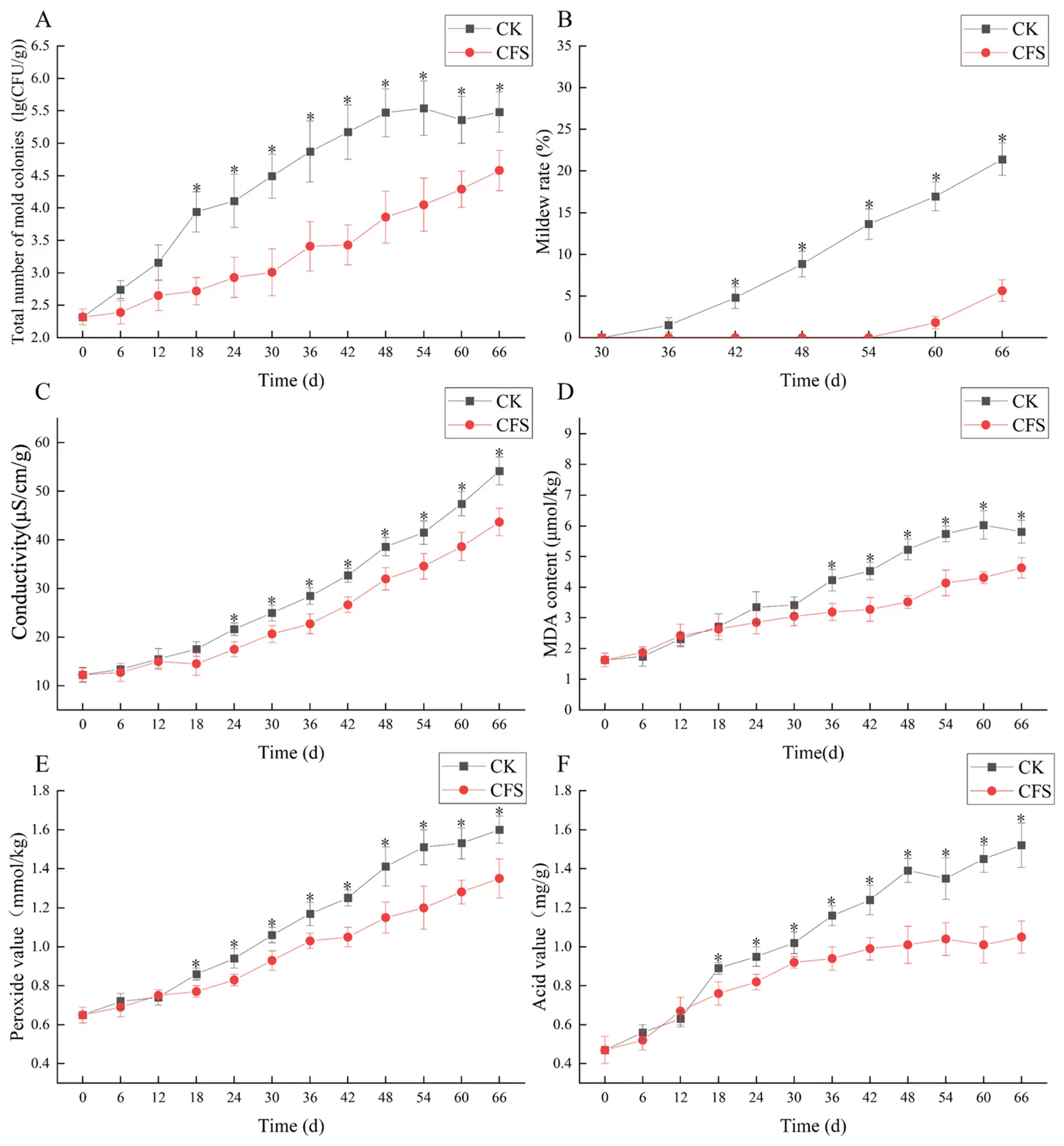
Effects of *P. pentosaceus* DB12 on total number of mold colonies (**A**), mildew rate (**B**), conductivity (**C**), MDA content (**D**), peroxide value (**E**) and acid value (**F**) of fresh peanuts during storage. Note: asterisks (*) indicate statistically significant differences (*p* < 0.05) between the CK and CFS groups.

Figure 2A shows the total number of mold colonies on fresh peanut surfaces. Mold loads rose gradually in both experimental groups over the entire storage period, and the CFS group maintained markedly lower colony numbers than the CK group from day 36 onward.

Figure 2B presents the mildew rate of fresh peanuts. Mold development was initially detected in the CK group at day 36, while the CFS group first exhibited mold growth on day 60. The CFS treatment postponed mold emergence by 24 days.

### 3.3. Conductivity and MDA Content

As illustrated in Figure 2C,D, both peanut groups exhibited increasing trends in conductivity and MDA content over the storage period. On the whole, both parameters in the CFS group were lower than those in the CK group. Statistically significant differences in conductivity emerged from day 24 onward, and MDA content in the CFS group was significantly lower than that in the CK group from day 36 of storage.

### 3.4. Alterations in Peroxide and Acid Values

As shown in Figure 2E,F, both the peroxide value and acid value increased over the storage period. At the initial stage of storage, no significant differences in these two quality indices were observed between fresh peanuts in the CK group and CFS group. From day 18 of storage onwards, the rate of increase in peroxide value accelerated markedly for the CK group, whereas a substantially lower rising rate was recorded in the CFS group. After 12 days, the acid value rose rapidly in the CK group, whereas the CFS-treated samples remained at a low level at the later storage stage.

### 3.5. Starch, Soluble Sugar and Sucrose Content

**Figure 3:**
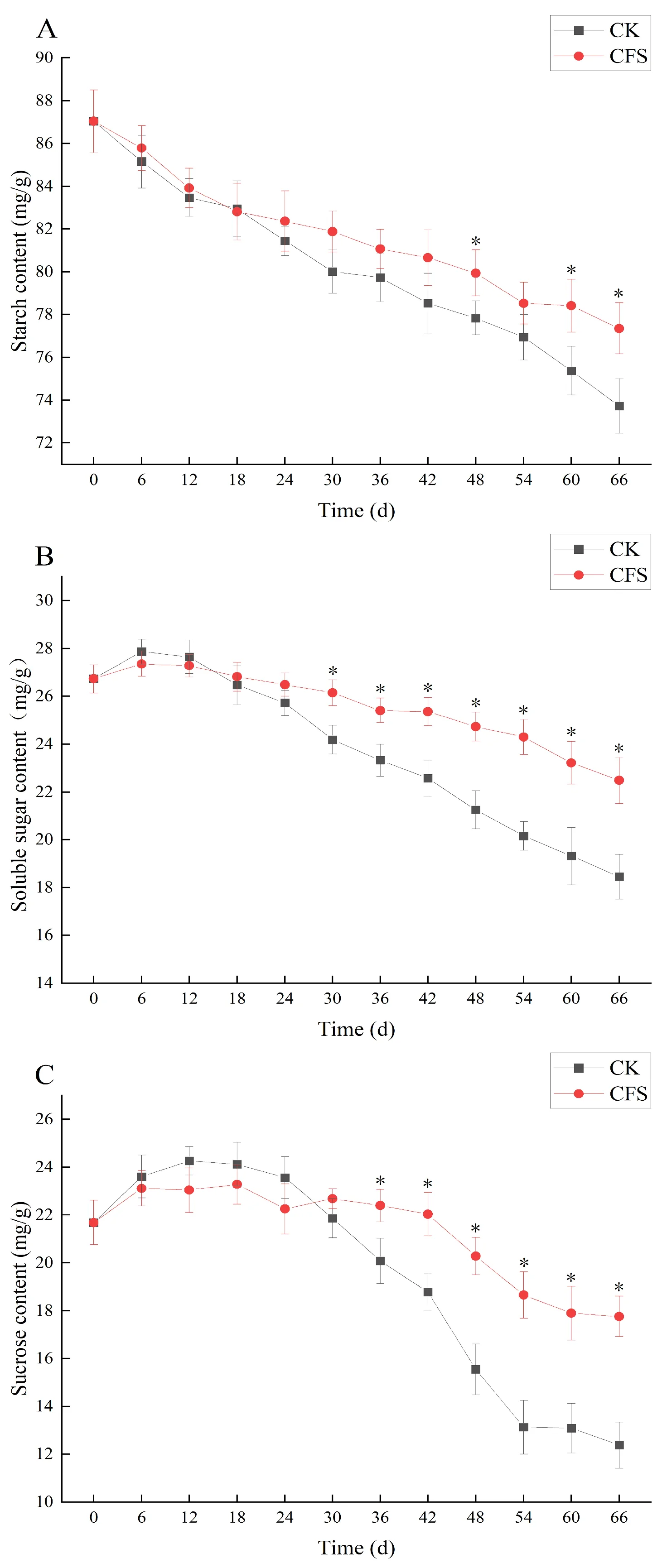
Effects of *P. pentosaceus* DB12 on starch content (**A**), soluble sugar content (**B**) and sucrose content (**C**) of fresh peanuts during storage. Note: asterisks (*) indicate statistically significant differences (*p* < 0.05) between the CK and CFS groups.

Starch content presented a decreasing tendency, as shown in Figure 3A. At the end of storage, the CFS group maintained markedly higher starch concentrations relative to the CK group. The soluble sugar and sucrose contents of fresh peanuts are shown in Figure 3B,C. Both parameters showed an initial temporary rise and then declined over storage. Importantly, soluble sugar and sucrose contents in the CFS group remained significantly higher than those in the CK group at all storage time points.

### 3.6. Metabolomic Analysis

**Figure 4:**
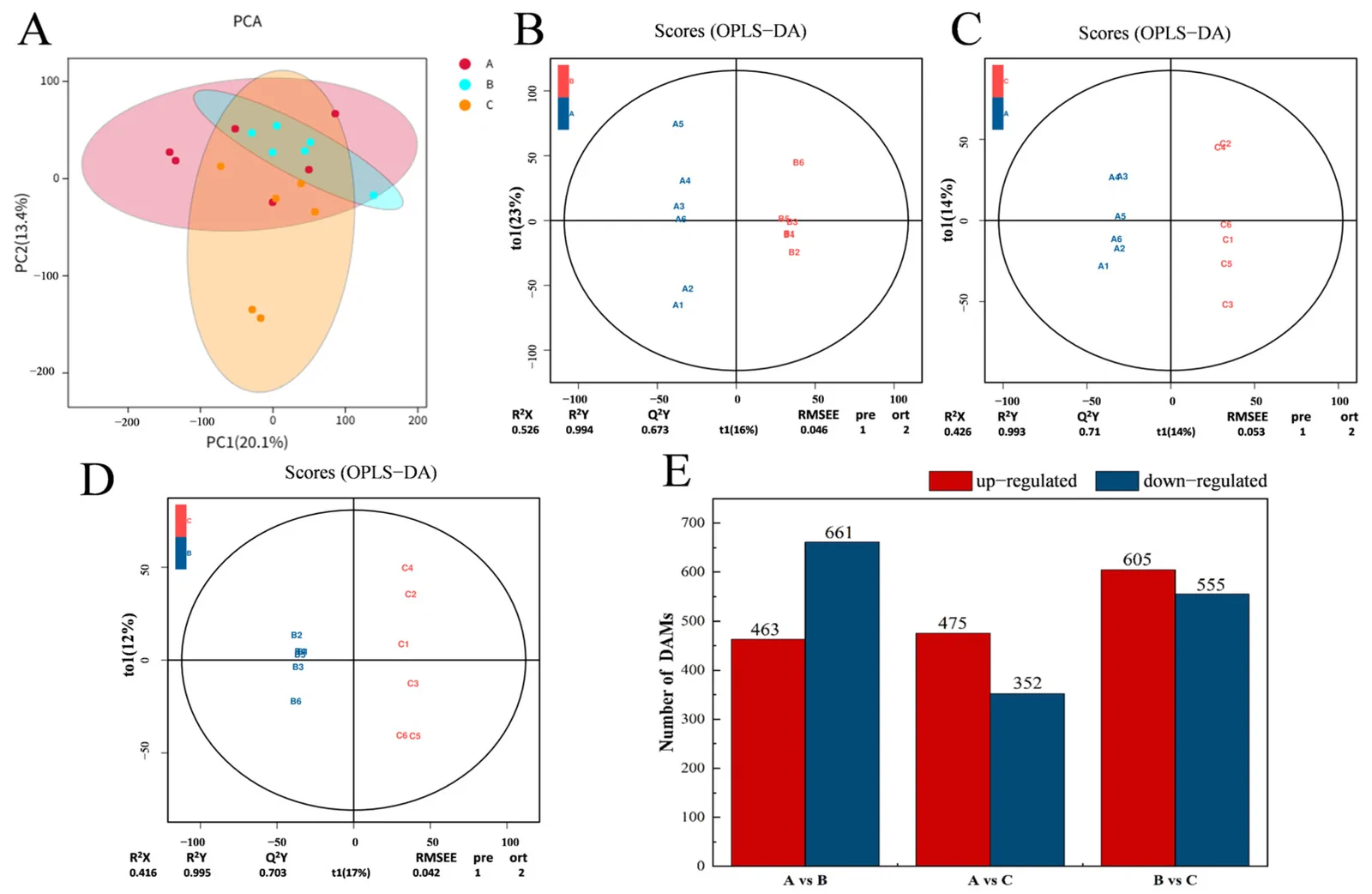
Metabolomic profiling of fresh peanuts. (**A**) PCA score plot of Group A (0 d), Group B (CK-24 d), and Group C (CFS-24 d). (**B**–**D**) OPLS-DA score plot for Group A vs. Group B, Group A vs. Group C, Group B vs. Group C, respectively. (**E**) Number of up- and down-regulated differentially accumulated metabolites (DAMs) in the three comparison groups. Each point represents an independent measurement, with distinct shapes and colors indicating different groups. A larger elliptical separation between sample clusters indicates greater metabolic divergence.

To elucidate the impact of *P. pentosaceus* DB12 CFS on internal metabolic alterations in stored fresh peanuts, an untargeted metabolomics approach based on LC-MS was employed to profile and identify metabolites. PCA was conducted to evaluate overall metabolic variations among treatment groups. As depicted in Figure 4A, a clear separation was observed between fresh peanuts from Group A (0 d), Group B (CK-24 d), and Group C (CFS-24 d), indicating significant metabolic divergence in *P. pentosaceus* DB12 CFS-treated peanuts after 24 days of storage, which warrants further investigation. Ellipses represent 95% confidence intervals.

**Figure 5:**
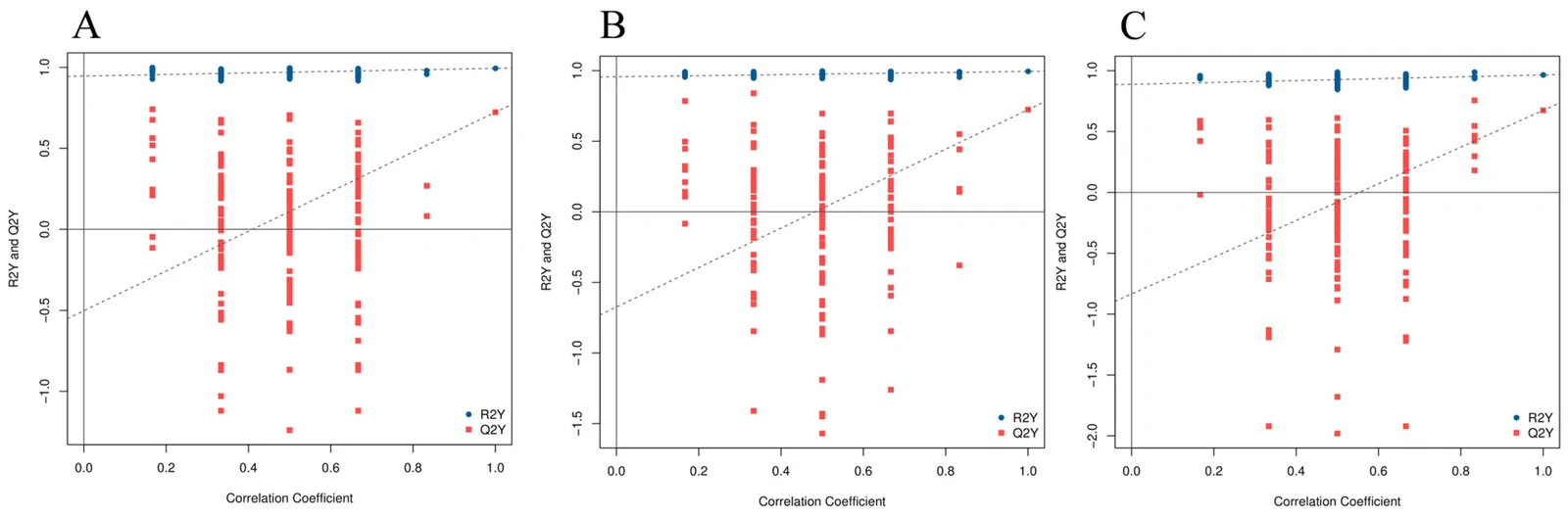
Permutation test results (*n* = 200) for OPLS-DA models of metabolite profiles. (**A**) Permutation test of the OPLS-DA models for Group A (0 d) vs. Group B (CK-24 d). (**B**) Permutation test of the OPLS-DA models for Group A (0 d) vs. Group C (CFS-24 d). (**C**) Permutation test of the OPLS-DA models for Group B (CK-24 d) vs. Group C (CFS-24 d). R^2^Y represents the explanatory ability of the model; Q^2^Y represents the predictive ability. The negative intercept of Q^2^Y indicates no obvious over-fitting of the OPLS-DA model.

To rigorously identify statistically significant differences among fresh peanut sample groups, orthogonal partial least squares-discriminant analysis (OPLS-DA) was performed. As shown in Figure 4B–D, samples from Group A (0 d), Group B (CK-24 d), and Group C (CFS-24 d) exhibited distinct separation along the predictive component, indicating the presence of discriminatory metabolites across treatment groups. To evaluate model robustness and rule-out over-fitting, permutation testing (*n* = 200) with 7-fold cross-validation was conducted. Group labels were randomly shuffled, and OPLS-DA models were reconstructed on permuted datasets to compute R^2^Y and Q^2^Y metrics iteratively. As shown in Figure 5, high R^2^Y values close to 1 were observed for all comparison groups, and Q^2^Y > 0.5. In addition, the intercept of Q^2^Y from permutation tests was below zero, demonstrating good robustness and reliable predictive power of the constructed OPLS-DA models. Differentially accumulated metabolites (DAMs) were defined as metabolites with VIP > 1 and *p*-value < 0.05 based on the OPLS-DA model. This strict screening process establishes a fundamental dataset for subsequent mechanistic exploration. As shown in Figure 4E, 1124 DAMs were identified in the Group A vs. Group B comparison group, among which 463 were up-regulated and 661 were down-regulated. A total of 827 DAMs were found in the Group A vs. Group C comparison group, consisting of 475 up-regulated and 352 down-regulated metabolites. Meanwhile, Group B vs. Group C comparison group yielded 1160 DAMs, including 605 up-regulated and 555 down-regulated metabolites.

**Figure 6:**
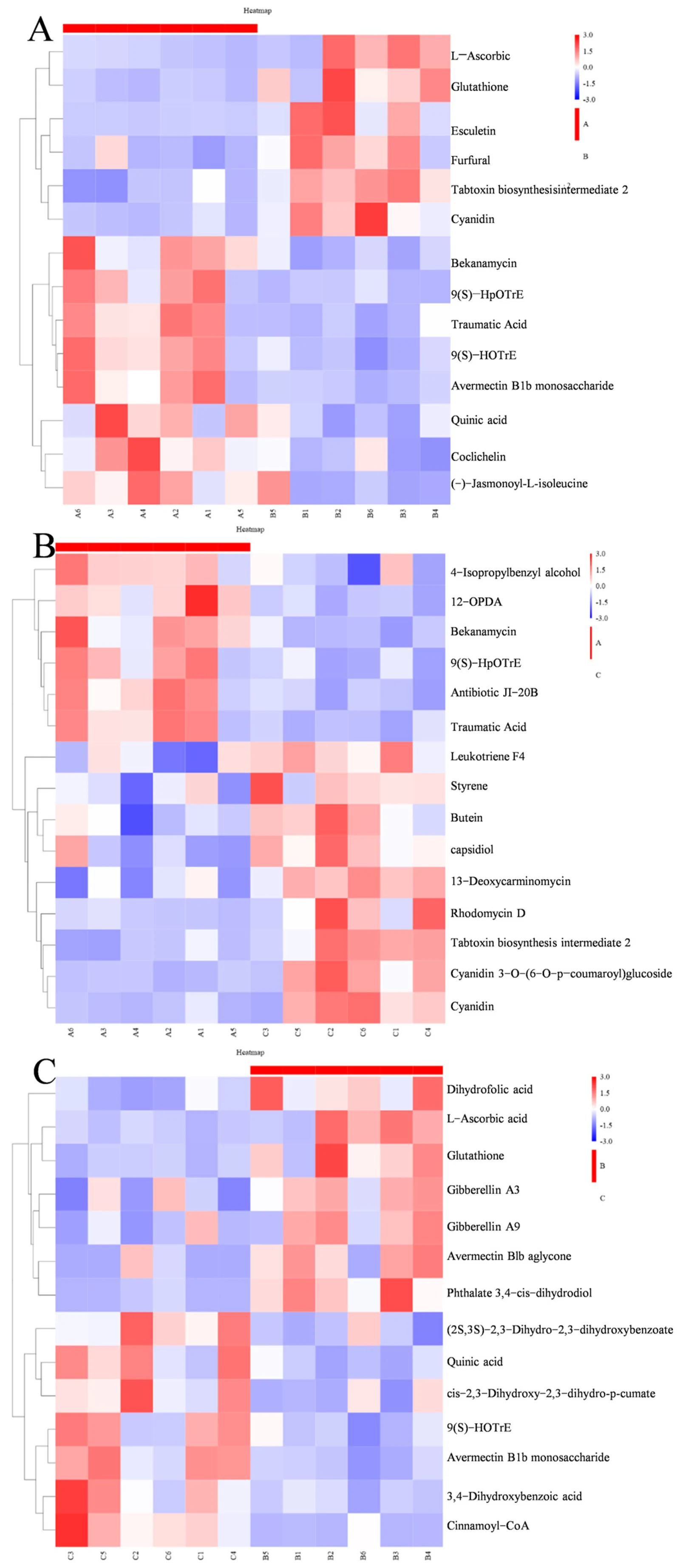
Clustered heatmap of disease resistance-associated DAMs in different groups. (**A**) Group A (0 d) vs. Group B (CK-24 d). (**B**) Group A (0 d) vs. Group C (CFS-24 d). (**C**) Group B (CK-24 d) vs. Group C (CFS-24 d). The color indicates the relative abundance of metabolites in the respective sample group, with the color bar in the lower right corner representing the trend in change. On the left side is the dendrogram of metabolite clustering, and on the right side is the name of the corresponding metabolite. The closer the two metabolite branches, the more similar their abundance levels. At the top is the dendrogram of sample clustering, and at the bottom is the sample name. The closer the branches of two samples, the more similar the overall metabolite profiles, indicating a more similar trend in metabolite abundance change.

Cluster analysis of DAMs combined with VIP scoring was conducted to characterize metabolic alterations in fresh peanuts during storage. Table S1 lists the DAMs identified in the Group A vs. Group B comparison group. DAMs related to disease resistance were selected from this dataset for subsequent analysis. Figure 6A displays a clustered heatmap of these DAMs, highlighting the significant up-regulation of L-ascorbic acid, glutathione, and esculetin, as well as the down-regulation of bekanamycin, traumatic acid, and quinic acid. Table S2 presents the DAMs identified in the Group A vs. Group C comparison group. From this dataset, disease resistance-associated DAMs annotated by KEGG pathways were systematically selected. Figure 6B illustrates a clustered heatmap of disease resistance-related DAMs in the Group A vs. Group C comparison group, revealing significant up-regulation of rhodomycin D, cyanidin, and styrene, along with down-regulation of 4-isopropylbenzyl alcohol, 12-OPDA and antibiotic JI-20B. Table S3 lists the DAMs screened from the Group B vs. Group C comparison group. Based on this dataset, DAMs associated with disease resistance functions were systematically screened via KEGG annotation. Figure 6C presents a clustered heatmap of disease resistance-associated DAMs in Group B vs. Group C comparison group, showing significant up-regulation of cis-2,3-dihydroxy-2,3-dihydro-p-cumate, cinnamoyl-CoA, and avermectin B1b monosaccharide, as well as down-regulation of gibberellin, dihydrofolic acid, and phthalate 3,4-cis-dihydrodiol.

**Figure 7:**
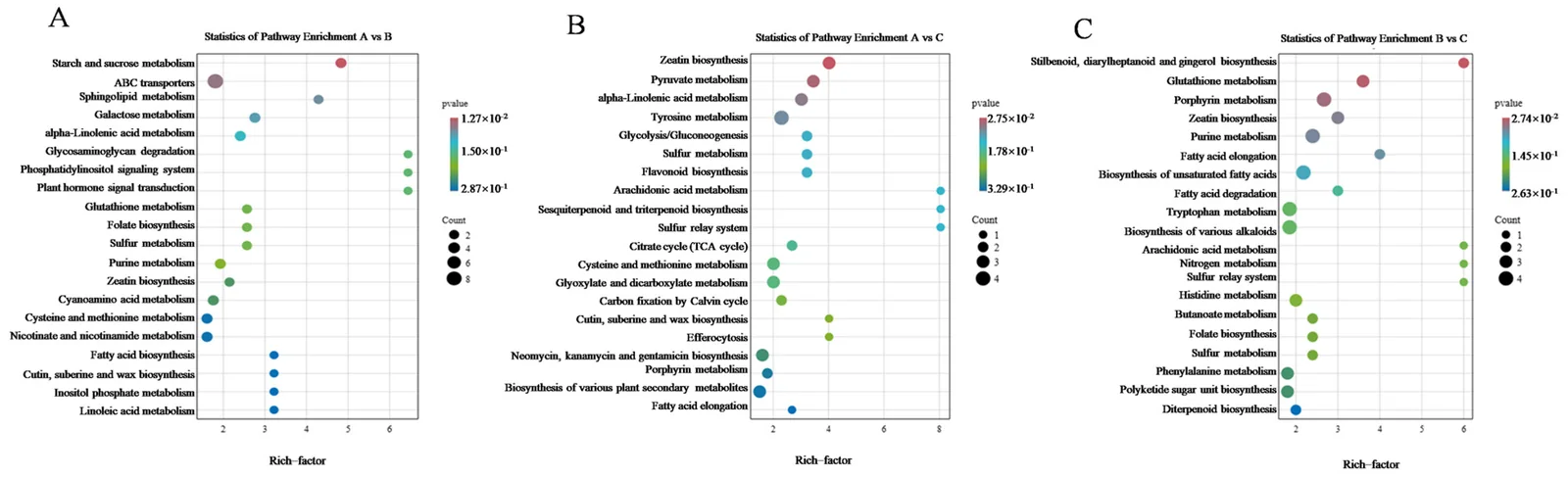
KEGG enrichment analysis of differential metabolites in different groups. (**A**) Group A (0 d) vs. Group B (CK-24 d). (**B**) Group A (0 d) vs. Group C (CFS-24 d). (**C**) Group B (CK-24 d) vs. Group C (CFS-24 d). The horizontal axis represents rich factor, indicating the relative enrichment strength of differential metabolites within specific pathways. The size of the bubble corresponds to the number of metabolites involved in each enriched pathway.

Metabolites were functionally annotated and classified based on their involvement in biological pathways using the KEGG database. Figure 7A–C present the KEGG pathway enrichment analysis for the three comparison groups, respectively. Notably, the sulfur relay system and zeatin biosynthesis pathway were significantly enriched across all three comparison groups, indicating their conserved roles in the metabolic response to storage conditions and CFS treatment. In the Group A vs. Group B comparison group (Figure 7A), significant pathway enrichments were observed for plant hormone signal transduction, ABC transporters and starch and sucrose metabolism, among other significantly altered pathways. For the Group A vs. Group C comparison group (Figure 7B), markedly enriched pathways contained flavonoid biosynthesis, arachidonic acid metabolism, and cysteine and methionine metabolism, together with other functionally related pathways. The Group B vs. Group C comparison group (Figure 7C) showed significant enrichment in the pathways of phenylalanine metabolism, diterpenoid biosynthesis, and zeatin biosynthesis. Collectively, these findings suggest that *P. pentosaceus* DB12 CFS may alleviate *A. flavus* infection and quality deterioration in stored fresh peanuts by activating biosynthetic pathways of key defense metabolites, including flavonoids, alkaloids, and diterpenoids.

## 4. Discussion

Fresh peanuts retain their native nutritional composition and exhibit unique sensory characteristics that are not achievable in dried counterparts, whether boiled or steamed. These characteristics deliver a distinctively sweet and umami-rich flavor profile [41]. Color, hardness, and weight loss rate are recognized as critical quality indicators for evaluating the shelf life and freshness of fresh peanuts. These parameters visually and effectively reflect product freshness and directly influence consumer purchasing behavior [42]. In this research, fresh peanuts and peanut kernels exhibited gradual deterioration in color, hardness, and weight loss rate during the entire storage period (Figure 1), consistent with the results of Wu et al. [5]. Moreover, *P. pentosaceus* DB12 CFS markedly retarded the browning, softening and weight loss of fresh peanuts and peanut kernels during postharvest storage (Figure 1). These results corroborate previous studies on LAB CFS applications in sweet corn and strawberry, indicating that *P. pentosaceus* DB12 CFS partially mitigates *A. flavus*-induced quality deterioration in fresh peanuts, thereby effectively preserving their commercial attributes [27,43].

Fresh peanuts, as high-moisture and highly perishable commodities, face significant challenges from aflatoxigenic *A. flavus* contamination during postharvest storage. Crucially, the proliferation of these toxigenic fungi markedly reduces the storage duration of fresh peanuts [44]. This study assessed the storage quality of fresh peanuts by detecting two core microbiological indicators, total number of mold colonies (representing microbial load) and mildew rate (indicating visible infection), so as to evaluate microbial proliferation and overt contamination levels throughout storage. A progressive increase in total number of mold colonies and mildew rate were observed during extended storage. However, *P. pentosaceus* DB12 CFS treatment significantly inhibited fungal proliferation on fresh peanut surfaces, as evidenced in Figure 2A,B. This inhibitory effect likely reflects the antimicrobial activity of *P. pentosaceus* DB12 CFS [45]. Alternatively, this protective effect may stem from *P. pentosaceus* DB12 CFS-triggered systemic resistance in fresh peanuts, which primes defensive responses to resist fungal colonization. Previous studies have reported that *Lactobacillus plantarum* CKXP13 and CWXP24 generate antimicrobial substances, such as proteinaceous metabolites and organic acids, that efficiently inhibit the growth of *Penicillium digitatum* on citrus fruits and exert prominent biocontrol efficacy [46]. Furthermore, existing studies have shown that *Lactobacillus pentosus* CW5 CFS markedly elevated the activities of superoxide dismutase (SOD), peroxidase (POD), and ascorbate peroxidase (APX) of kiwifruit, reducing oxidative stress and delaying senescence progression during storage [28]. Integrating metabolomic profiling (Figure 7C), *P. pentosaceus* DB12 CFS likely enhances resistance against fungal infection in stored fresh peanuts by activating key defense-related metabolic pathways, including phenylalanine metabolism, stilbenoid biosynthesis and diarylheptanoid biosynthesis, thereby strengthening biochemical defenses during storage. Similarly, *Lactobacillus plantarum* O_2_ enhances resistance against *Phytophthora capsici* in pepper plants by elevating the activities of key enzymes, including peroxidase, polyphenol oxidase, and phenylalanine ammonia-lyase [47].

Conductivity measurements serve as well-established biomarkers for quantifying membrane permeability in biological systems [48]. Meanwhile, MDA content acts as a biochemical marker for plant senescence and membrane deterioration [49]. This study monitored membrane deterioration in stored fresh peanuts using these two complementary biomarkers. Both conductivity and MDA content exhibited progressive increases during storage (Figure 2C,D), which may be attributed to *A. flavus* infection causing damage to the cell membranes of fresh peanuts [50]. Importantly, the CFS group presented remarkably reduced conductivity and MDA accumulation relative to the CK group, indicating that *P. pentosaceus* DB12 CFS effectively alleviates oxidative membrane damage in fresh peanuts triggered by *A. flavus* (Figure 2C,D). Integrating metabolomic profiling, *P. pentosaceus* DB12 CFS activates the glutathione metabolism and phenylalanine metabolism pathways in fresh peanuts. Such metabolic activation promotes the biosynthesis of glutathione, lignin polymers, and flavonoid compounds, which synergistically alleviates oxidative damage via reactive oxygen species (ROS) elimination and strengthens cell walls to resist pathogenic invasion. Similarly, the application of *Meyerozyma caribbica* as a biocontrol agent increases the production of secondary metabolites, including total phenolics, flavonoids, and lignin polymers in kiwifruit, thereby effectively reducing MDA accumulation by 38% during storage [51].

As an oil-rich commodity, peanut kernels contain approximately 80% unsaturated fatty acids in their oil fraction, making them highly susceptible to oxidative degradation [52]. Peroxide and acid values are recognized as critical indicators of oil quality and oxidative deterioration. Therefore, this study monitored lipid oxidation and hydrolytic rancidity in fresh peanuts by detecting dynamic variations in peroxide value and acid value during the entire storage period. Continuous increases in peroxide and acid values were detected in peanut oil during long-term storage (Figure 2E,F), which may result from *A. flavus* colonization accelerating unsaturated fatty acid oxidation [53]. Crucially, the CFS-treated group exhibited significantly lower increases in both peroxide and acid values compared to the CK group (Figure 2E,F). Integrating metabolomic profiling, *P. pentosaceus* DB12 CFS activates the stilbenoid biosynthesis pathway in fresh peanuts, inducing the production of flavonoids and stilbenoid compounds. These metabolites confer significant resistance against *A. flavus*-induced oxidative rancidity in peanut lipids [54]. Conversely, Lee et al. reported that kimchi-derived strains *Latilactobacillus curvatus* WiKim38, *Lactococcus lactis* WiKim0124 and *Companilactobacillus allii* WiKim39 exhibit pro-oxidative effects on lipids [55]. This highlights the distinct protective role of *P. pentosaceus* DB12 CFS in retarding oxidative rancidity in fresh peanut lipids resulting from *A. flavus* contamination.

Soluble sugars and sucrose constitute core determinants of peanut flavor quality, with their concentrations directly influencing sweetness perception, flavor complexity development, and overall sensory acceptability [56]. This study evaluated the impact of *P. pentosaceus* DB12 CFS on flavor quality retention in stored fresh peanuts by analyzing the dynamics of starch, soluble sugar and sucrose contents throughout the storage period. The results showed that, as illustrated in Figure 3A, starch content remained relatively stable and did not exhibit significant changes during storage. However, soluble sugar and sucrose contents in fresh peanuts initially increased slightly before progressively declining during storage (Figure 3B,C). This metabolic shift may be attributed to *A. flavus* infection, which accelerates carbohydrate consumption within peanut tissues during fungal colonization [12]. Notably, as shown by the data presented in Figure 3B,C, the decline rate of soluble sugars and sucrose was markedly lower for the CFS-treated group relative to the CK group. This demonstrates that *P. pentosaceus* DB12 CFS effectively delays the accelerated depletion of soluble sugars and sucrose caused by *A. flavus* infection, thereby enhancing the preservation of flavor attributes in stored fresh peanuts.

As illustrated in Figure 4A–E, *P. pentosaceus* DB12 CFS treatment induced distinct metabolic alterations in fresh peanuts during storage, with notable changes in key disease-resistance metabolites (Figure 6A–C). Notably, quinic acid, cinnamoyl-CoA, and 3,4-dihydroxybenzoic acid—critical intermediates in phenylalanine metabolism—were consistently upregulated (Figure 6C). Figure 7A–C demonstrate that *P. pentosaceus* DB12 CFS treatment altered KEGG pathway enrichment in fresh peanuts, with significant enrichment observed in disease resistance-associated pathways, including phenylalanine metabolism, stilbenoid biosynthesis, diarylheptanoid and gingerol biosynthesis, and glutathione metabolism (Figure 7C). Phenylalanine functions as a central hub metabolite in plant systems and serves as the primary precursor for multiple critical biotransformation pathways. Ramzan et al. showed that exogenous phenylalanine can effectively alleviate the effects of drought stress in mustard (Brassica juncea) through boosting enzymatic and nonenzymatic antioxidant systems, hence promoting ROS detoxification [57]. Furthermore, studies reveal that *Stylosanthes guianensis* subjected to manganese hyperaccumulation stress activates its antioxidant defense system through upregulation of phenylpropanoid and flavonoid metabolic pathways. Compared to untreated controls, flavonoid metabolites significantly increased, leading to enhanced shoot and root biomass production for Mn detoxification [58]. In research on rice resistance to *Tilletia horrida*, investigators discovered that keratin, corky base and wax biosynthesis, as well as flavonoid biosynthesis, form core pathways responsible for resistance to this pathogen [59].

## 5. Conclusions

This study investigated the efficacy of *P. pentosaceus* DB12 CFS in controlling postharvest *A. flavus* contamination in fresh peanuts. Results demonstrate that *P. pentosaceus* DB12 CFS effectively mitigates *A. flavus*-induced deterioration of key quality parameters in fresh peanuts, including color stability, textural hardness, and weight retention during storage. It markedly lowers the total mold colony count and mildew rate, relieves oxidative damage to cell membranes, slows oxidative rancidity in peanut oil, and reduces the degradation of starch, soluble sugars and sucrose induced by *A. flavus.* Metabolomics data indicate that *P. pentosaceus* DB12 CFS activates key metabolic pathways in fresh peanuts, including phenylalanine metabolism, stilbenoid biosynthesis, diarylheptanoid and gingerol biosynthesis, and glutathione metabolism, thereby delaying the deterioration associated with *A. flavus* infection. This research underscores the potential of *P. pentosaceus* DB12 CFS to control postharvest *A. flavus* contamination in fresh peanuts, establishing a basis for creating safe and effective quality preservation strategies.

## Data Availability

The original contributions presented in this study are included in the article and Supplementary Materials. Further inquiries can be directed to the corresponding author.

## Supplementary Materials

The following supporting information can be downloaded at: https://www.mdpi.com/article/10.3390/foods15213939/s1, Table S1: Differentially accumulated metabolites (DAMs) identified in fresh peanuts of Group A (0 d) vs. Group B (CK-24 d) comparison group; Table S2: Differentially accumulated metabolites (DAMs) identified in fresh peanuts of Group A (0 d) vs. Group C (CFS-24 d) comparison group; Table S3: Differentially accumulated metabolites (DAMs) identified in fresh peanuts of Group B (CK-24 d) vs. Group C (CFS-24 d) comparison group; Table S4: Annotation confidence and supporting parameters for Differentially accumulated metabolites (DAMs).
